# In Stent Restenosis Predictors after Carotid Artery Stenting

**DOI:** 10.4061/2010/864724

**Published:** 2010-03-14

**Authors:** Mirco Cosottini, Maria Chiara Michelassi, Walter Bencivelli, Guido Lazzarotti, Silvia Picchietti, Giovanni Orlandi, Giuliano Parenti, Michele Puglioli

**Affiliations:** ^1^Department of Neuroscience, University of Pisa, Pisa 56100, Italy; ^2^Service of Neuroradiology AOU, Pisa 56100, Italy; ^3^Department of Radiology, University of Pisa, Pisa 56100, Italy; ^4^Department of Internal Medicine, University of Pisa, Pisa 56100, Italy

## Abstract

*Purpose*. The long-term efficacy of carotid artery stenting is debated. Predictors of stent restenosis are not fully investigated. Our aim was to assess the incidence of long term restenosis after CAS and to identify some predictors of restenosis. *Methods*. We retrospectively selected 189 treated patients and we obtained the survival Kaplan-Meier curves for overall survival, for freedom from stroke or death and from restenosis. To correlate clinical, radiological, and procedural variables to stent restenosis, an univariate analysis was performed while to determine independent predictors of restenosis, a multivariate analysis was applied. *Results*. At 1, 3, and 5 years, the cumulative overall survival rate was 98%, 94%, and 92% with a cumulative primary patency rate of 87%, 82.5%, and 82.5%. The percentage residual stenosis after CAS and multiple stents deployment were independent predictors of restenosis, while diabetes and tumors are suggestive but not significant predictors of restenosis. *Conclusions*. In our CAS experience, encouraging long-term results seem to derive from both neurological event free rate and restenosis incidence. Adequate recanalization of the treated vessel is important to limit the development of stent restenosis. Multiple stents deployment, and with less evidence, diabetes, or neoplasms has to be considered to facilitate restenosis.

## 1. Introduction

Based upon data of multicenter trials, carotid endarterectomy (CEA) is a proven treatment in carotid artery stenosis and it is considered the most effective method to prevent stroke occurrence in patients with symptomatic and asymptomatic high-grade carotid artery disease [1, 2]. For high risk surgical patients, percutaneous carotid transluminalangioplasty and stenting (CAS) are emerging with encouraging results as alternative method to carotid endarterectomy [3]. 

The endovascular treatment of carotid artery stenosis with CAS has an acceptable periprocedural complication rate and stroke/death rate at one year especially with the use of cerebral protection devices [4–6]. On the basis of randomized [3] and non randomized [7] multicenter trials results, in high surgical risk patients, protected CAS is actually considered equal to surgery in the hands of experienced operators [8].

The efficacy of CAS over time is under clinical evaluation. Data regarding efficacy of CAS over a longer period of time (until five years) are recently emerging [9]. In a recent meta-analysis [10] the early restenosis rates after CAS compare well with those reported for CEA, nevertheless due to the short followup period of many published works the long term durability of CAS needs of further studies. Moreover few information are available about independent clinical, radiological or procedural predictors of restenosis after CAS in patients with long followup.

The aim of our study was to assess the incidence of long term in-stent restenosis after CAS and to identify some clinical, radiological or procedural predictors of restenosis.

## 2. Materials and Methods

### 2.1. Patients Selection and Data Collection

We conduct a retrospective study including patients that underwent CAS in our centre from 1997 to 2004. Out of 400 procedures we select from our data base 189 patients that underwent to a complete clinical and CD-US followup exclusively in our centre avoiding to include patients that were followed from other hospitals and operators. Previously, we have obtained for this study the approval from our Istitutional Review Board. Indications to CAS were ICA stenosis higher than 70% in symptomatic patients and asymptomatic patients with one of the following co-morbidities: two or more coronary vessels with >70% stenosis, an ejection fraction <30%, bronchopulmonary obstructive disease, recurrent stenosis after CEA, previous radical neck surgery or radiation therapy, surgically inaccessible lesions and controlateral carotid artery occlusion. Diagnosis of ICA stenosis was made by CD-US and by MRA or CTA as a confirmatory test and definitively stated with DSA.

One hundred forty eight patients were males and 41 females with a mean age of 72.31 ± 8.17 (range 37–87 y). 200 vessels were treated with CAS (in 11 patients (5.8%) a bilateral treatment was performed). In 169 vessels the carotid stenosis was atherosclerotic (84.5%), in 23 it was postsurgical (restenosis after endarterectomy) (11.5%) and in 8 post radiotherapy (4%). Ninety eight vessels (49%) did not cause neurological ipsilateral symptoms while 102 (51%) did it.

Clinical, radiological and procedural data supposed to interfere with in-stent restenosis were collected. Clinical variables included age, sex, symptomatic status of patient, and presence of supposed risk factors and comorbidity (smoke, chronic pulmonary diseases, hypertension, diabetes, hypercholesterolemia, coronary arterial disease, neoplasms). Radiological variables were obtained with the evaluation of pre and post-procedural diagnostic DSA. They included the grade of stenosis, presence of ulcerated plaques, nature of stenosis (atherosclerotic versus not atherosclerotic), percentage of residual stenosis after CAS. Procedural variables included postdilatation, type of stent, number of deployed stents.

### 2.2. CAS Procedure

All procedures were performed with local anesthesia and percutaneous transfemoral access F8.

Patients were premedicated with aspirin (100 mg/die) and ticlopidine (500 mg die) at least three days preintervention. 

The patients received intra-arterial administration of 70 IU/Kg of heparin to achieve an activated clotting time (ACT) longer than 200–250 sec. By using a 100-cm long guiding catheter, the filter guidewire was introduced crossing the stenosis and the cerebral protection device, a self-expanding basket type filter, was deployed in the cervical portion of internal carotid artery. Out of 200 procedures, 69 (34.5%) were performed with a cerebral protection device (Filter Wire EZ Boston Scientific). A self-expandable stent was mounted on the protection device guidewire and placed and deployed across the stenosis. In our study group the type of stent in the 200 procedure was: 132 (66%) Carotid WallStent Monorail (*Boston Scientific*), 39 (19.5%) Precise Stent (*Cordis Corp., Johnson & Johnson Company*), 17 (8.5%) Easy WallStent (*Boston Scientific*), 7 (3.5%) Smart Stent (*Cordis Corp., Johnson & Johnson Company*), 3 (1.5%) Wallstent* (Boston Scientific Corp.*), 1 (0.5%) Acculink (*Guidant Corp*), 1 (0.5%) Omnilink (*Guidant Corp*). The stent was dilated to reach an adequate vessel recanalization by using an appropriate size angioplasty balloon. One mg of atropine sulphate was intravenously administered during angioplasty balloon insufflations to prevent carotid sinus stimulation and bradycardia. Then the cerebral protection device, when used, was removed. Predilation with a 3 mm PTA balloon catheter (generally Ultrasoft 5 V Boston Scientific Natick MA USA) was performed in 34 patients before stent placement in tight stenoses with a residual lumen smaller than the diameter of the stent delivery catheter. 

In 19 patients the misplacement of the first stent necessitates of the deployment of a further stent.

A permanent daily medication with acetylsalicylic acid (100 mg) and a prevention therapy with 500 mg/die of ticlopidine was started for one month, after endovascular recanalization. 

Before the interventional procedure, all patients were submitted to diagnostic DSA by selective injection of both common carotid arteries and vertebral arteries of at least one side. Intracranial vessels and carotid bifurcation evaluation of the treated side was repeated after endovascular procedure.

In case of unprotected CAS, the procedure differed exclusively for use of guidewire instead of filter guidewire to encompass the stenosis. Patients remained in a monitored setting overnight and discharged in the following day.

### 2.3. CD-US Follow-Up

All patients were followed with CD-US examination at 24 hours, 1, 3, 6 months after the procedure and every 6 months thereafter. In each diagnostic session a clinical interview was made. The restenosis detection was based on CD-US by using modified velocity criteria of Washington University [11] developed in our centre and validated with digital subtraction angiography. Moreover B-mode imaging of the arterial lumen and spectral waveform analysis were used to assess possible restenosis occurrence. Elevations in peak systolic velocity or ICA/CCA ratio with respect to the first post procedural CD-US examination were considered as suggestive of progressive in-stent restenosis. A peak systolic velocity higher than 220 cm/sec was interpreted as a stenosis higher than 50%. 

A in stent stenosis higher than 50% was considered a restenosis.

### 2.4. Statistical Analysis

Continuous data are presented as mean value ± SD, and categorical data are presented as frequencies. 

Overall survival curves (Kaplan-Meier) were obtained using as end point, respectively, death, occurrence of major related event (death and stroke) and occurrence of restenosis. Patients dead for nonrelated causes were considered as lost to the observation.

Clinical, radiological and procedural variables were used as group variables in an univariate survival analysis (Kaplan-Meier, log-rank Mantel Cox test for significance of difference). This analysis was performed by patient (*n* = 189) relative to the clinical variables and by vessel (*n* = 200) relative to the radiological and procedural variables.

Finally a multivariate survival analysis was performed by using the proportional hazard stepwise Cox model (*P* to enter = .15). This analysis was performed by patient and excluding the single patient bilaterally treated with a single vessel restenosis (*n* = 188). Statistical analysis was performed with Stat View 5.02 software package (Abacus Concepts).

## 3. Results

All patients were observed for a mean of 29.9 months (range 0–99) median 26 months. The overall periprocedural complications were 5 (2.6%): 2 fatal strokes (1.05%), 1 major stroke (0.5%) and 2 minor strokes (1.05%). 

**Figure 1:**
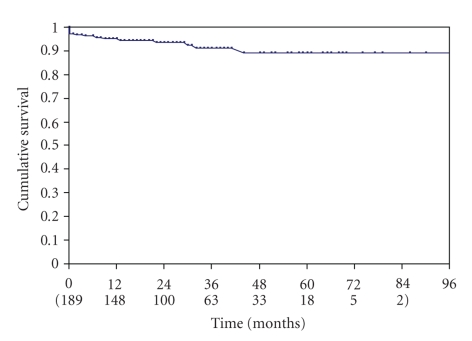
Kaplan Meyer analysis curve of the cumulative freedom from stroke and death.

The overall survival rate was 98% at 1 year, 94% at 3, and 92% at 5 years. The freedom from stroke and death defined as the freedom from all ipsilateral strokes and related deaths, was 95.1% at one year, 91.4% at 3 years and 89.1% at five years from treatment (Figure 1). During the followup we observed 5 periprocedural complications and 9 further strokes (4.8%), all of them were homolateral to the treated vessel. Out of 9 patients with stroke, 7 deceased (3.7%). 

**Figure 2:**
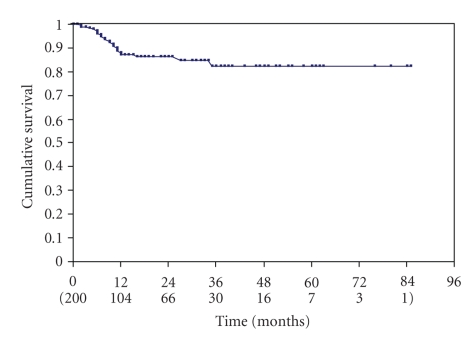
Kaplan Meyer analysis curve of the cumulative freedom from restenosis.

Twenty-three patients for a total of 23 treated vessels (23/200, 11.5%) developed a in-stent restenosis. Six restenosis occurred after stenting of 31 not-atherosclerotic plaques while 17 restenosis followed the stenting of 169 atherosclerotic stenosis. Restenosis occurred proximally to the stent implantation (common carotid artery) in four vessels, distally to the stent implantation (internal carotid artery) in one, and in the middle segment of the stent in 18 vessels. The grading of the restenosis was moderate (50%–79%) in 18 vessels (18/200, 9%), and severe (>80%) in 5 vessels (5/200, 2.5%). Only three patients (3/23, 12%) had a neurological event homolateral to the treated and restenosed vessel. The remaining patients with restenosis were asymptomatic. The cumulative rate of freedom from restenosis was, respectively, of 87%, 82.5% and 82.5%, respectively, at 1, 3 and 5 years (Figure 2).

Out of 23 restenosis, 10 were retreated with angioplasty alone (7 restenosis) or combined angioplasty and stenting (3 restenosis). In one patient with restenosis the intention to treat failed for the highly deformed stent that did not permit the crossing of the stenosis with the angioplasty balloon catheter. In three cases the treatment of restenosis was performed with a cerebral protection device placement. No periprocedural complications occurred in all retreated patients.

**Figure 3:**
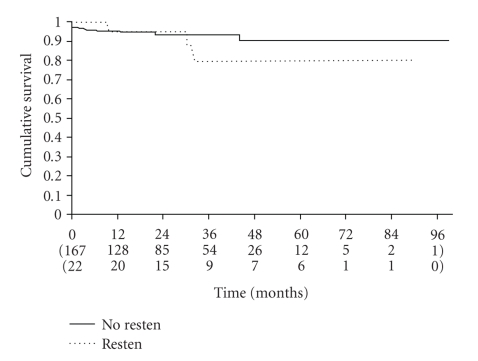
Kaplan Meyer analysis curves of the cumulative freedom from stroke and death in patients with (dotted line) and without (continuous line) restenosis.

The cumulative rate of survival did not differ from patients with restenosis with respect to patients without it (*P* = .48). The comparison of the Kaplan-Meyer curves in patients with and without restenosis does not reveal a significant difference (*P* = .37) in the free neurological event rate (Figure 3) that was 95%, 80% and 80% in the first group with respect to 95%, 93% and 90% in the latter, respectively at 1, 3 and 5 years.

Out of all the clinical radiological and procedural variables, the univariate analysis revealed that residual stenosis after stenting and the number of deployed stents for lesion are the only variables that correlate with the restenosis occurrence (*P* = .0007 and *P* = .04, resp.).

Multivariate analysis showed that the residual stenosis after stenting is a positive predictor of restenosis with relative hazard 1.091 per percent unit of residual stenosis, (CI 95% 1.050–1.130) *P* < .0001.

Another significant predictors was double stent deployment with relative hazard 5.2, (CI 95% 1.49–18.5) *P* = .0084. Suggestive but not significant variables, included in the stepwise model, were diabetes with relative hazard 2.30, *P* value = .070 and neoplasms with relative hazard 2.53, *P* value = .085.

## 4. Discussion

The overall periprocedural complications in our series are comparable to the literature results [4] and within the range of the acceptable perioperative risks after CEA [12].

The stroke or death rate (4.9% at one year, 8.6% at 3 years and 10.9% at five years from treatment) is in line with former publications that report 4%–7% at one year [9, 13, 14], 10.1%–11% at 3 years [9, 15] and 15.1% at 5 years [9] indicating encouraging prospective for CAS also over a long period of time.

The overall incidence of restenosis >50% is 11.5% and it is comparable to the value reported for CEA (10%) at one year [16] and lower to 20% at one year of angioplasty alone [17] confirming the competitiveness of CAS and CEA and the improvement of angioplasty durability after a stent deployment. Our cumulative restenosis rate at one and two years is 13 and 13.8%, respectively, slightly higher to the values derived from a recent meta-analysis (6 and 7.5% at one and two years) [10] but within the range reported in the literature varying from 0.6% [18] to 20.8% [19]. If we consider a significant restenosis as higher to 80%, we obtain an overall incidence of restenosis of 2.5% that is lower to the 4% reported in the aforementioned meta-analysis study [10]. Lower restenosis rate are reported in the single long-term multi-centre study of ELOCA registry [9] (1%, 2% and 3.4%, resp. to 1, 3 and 5 years). The variable incidence of restenosis in the different centres means that restenosis is not however negligible and it induce us to monitor the patients submitted to CAS over time. 

We outline that the Kaplan-Meyer analysis for survived and free from neurological events does not differ between patients with and without restenosis confirming the benign course of in-stent restenosis [20] as occur for restenosis after CEA [21]. Although there are few and often asymptomatic significant (>80%) in-stent restenosis, probably they will increase in the next time and may became, in future, a clinical problem due to the widespread application of CAS [22]. Concerning to the treatment of in-stent restenosis, angioplasty has been recently recommended as the primary approach to hyperplastic lesions with repeat stenting in cases of suboptimal results [23]. Although further experiences in carotid artery restenosis after stent are necessary to formulate standardized approaches, in our limited experience, the endovascular treatment of restenosis seems to be safety and when it is not feasible, surgical treatment can be considered [24]. 

According to previous studies, restenosis occurs preferentially in the first year from the endovascular procedure (20 out of 23 in the first 12 months) and reduces its prevalence in the subsequent years. It is generally accepted that the major cause of restenosis and of its benign course is neointimal hyperplasia with smooth muscle cells proliferation [25] that prevailed in the first 12 months after CEA [26].

Specific risk factors in the development of restenosis after CAS remains to be elucidated: some studies have identified advanced age [27, 28], hyperglycemia [27, 29], smoke [27] and previous CEA [30]. In our study group, out of all the evaluated clinical radiological and procedural variables, multivariate analysis reveal that the residual stenosis after CAS is a stronger predictor of restenosis with a consistent increased risk of restenosis (1.091 per % unit of residual stenosis). Suboptimal technical results after CAS was previously described as clinical predictor of restenosis in the first six months of followup [31] and a small post procedural stent dimension evaluated with intravascular ultrasound imaging has been demonstrated to be associated with a higher risk of restenosis also in carotid artery stenting [32]. Although larger studies with long term followup do not reveal [33] this radiological variable as predictor, recently suboptimal result with residual stenosis has been reported as a restenosis predictor [34] Carotid neointimal proliferation and stent auto-expansion have counteracting effects: the first one predominates during the first year after stenting, whereas the latter in the subsequent year [35]. We may suppose that in some cases, unexpanded carotid stent could imbalance these complex counteracting effects in favour of neointimal proliferation inducing post procedural stenosis and a following long term restenosis.

Our results suggest that post procedural stenosis has a higher risk of restenosis and it conflicts with the opinion that to pursue a perfect angiographic result after CAS is not necessary [36]. Nevertheless, small post procedural lumen dimension as a restenosis predictor have to be considered with caution and have not to induce an opposite aggressive behaviour because it is known that high post dilation pressures increase the risk of embolization and neointimal proliferation [37]. 

To our knowledge the implantation of multiple stents as a restenosis predictor after CAS has been mentioned in a brief report [28]. In our experience, similarly to the results of the coronary stenting, by both univariate [38] and multiple logistic regression analysis [39] the number of stents per lesion is a significant variable and independent predictor of restenosis. Probably the higher risk of in-stent restenosis may be due to a larger surface area covered with stent material or to the overlapped edges of the stent inducing a more enunciated intimal hyperplasia. 

Our study identify other clinical variables with a less relevant influence on restenosis such as diabetes and neoplasms. The role of diabetes has been described as a predictor of carotid [27, 28] and coronary in-stent restenosis [40] and may be due to smooth muscle cell proliferation common in diabetic patients [41]. The role of neoplasms in the restenosis occurrence is prone to speculative interpretations, in fact a series of cell proliferation regulatory pathways have been associated with plaque progression, stenosis and restenosis after angioplasty as well as in cancer progression [42]. 

The main limitations of the study are the small number of patients and events sourcing by a single centre study. However the detection of independent predictors after CAS that evoke the predictors after coronary stenting [43], may be useful to be largely studied in prospective randomized trials.

In our CAS experience encouraging long term results seem to derive from both neurological event free rate and restenosis incidence. Because of residual stenosis after CAS is a strongest independent predictor of restenosis, adequate recanalization of the treated vessel seems an important goal to limit the development of restenosis. Multiple stents deployment and with less evidence, diabetes or neoplasm have to be considered to facilitate in-stent restenosis after CAS.
